# Appendiceal intussusception due to endometriosis: a case report

**DOI:** 10.1093/jscr/rjag267

**Published:** 2026-04-15

**Authors:** Christine Holloway, Camryn Butera, Taylor Bronson, Justin Spatar

**Affiliations:** General Surgery, Regional Hospital of Scranton, Scranton, PA 18510, United States; MS3, Philadelphia College of Osteopathic Medicine, Philadelphia, PA 19131, United States; MS3, Touro College of Osteopathic Medicine, Middletown, NY 10940, United States; MS3, Philadelphia College of Osteopathic Medicine, Philadelphia, PA 19131, United States

**Keywords:** appendiceal intussusception, endometriosis, appendectomy

## Abstract

The rate of implantation of endometrial tissue onto the appendix varies greatly within the literature with appendiceal intussusception being a rare clinical presentation in this small subset of patients. Here we discuss a case of appendiceal endometriosis with robotic partial cecectomy and appendectomy. This report describes our experience with appendiceal cecal intussusception. We report a case of appendiceal endometriosis causing intussusception. It is important to consider endometriosis as a cause of appendiceal intussusception in the differential diagnosis of females presenting with the common chief complaint of abdominal pain, especially in the setting of prior gynecological surgeries.

## Introduction

Intussusception is defined as the invagination of the proximal aspect of the bowel into the distal aspect of the bowel [1]. Appendiceal intussusception is an extremely rare presentation, especially with endometrial tissue implantation being the lead point. Reported by Allahqoli *et al.* endometriosis of the appendix has been observed in only 2.67% of women who presented with acute appendicitis and 7.23% of cases as an incidental finding during gynecological surgeries [2]. Intussusception of the appendix as the presentation of this appendiceal endometriosis is very uncommon with Collins reporting the incidence as 0.01% [3]. Appendiceal endometriosis presentation varies from acute appendicitis to asymptomatic, making preoperative diagnosis very difficult. We report a case of a female patient presenting with an asymptomatic inverted appendix on colonoscopy that underwent robotic partial cecectomy. Subsequent histopathology revealed appendiceal endometriosis.

## Case presentation

**Figure 1:**
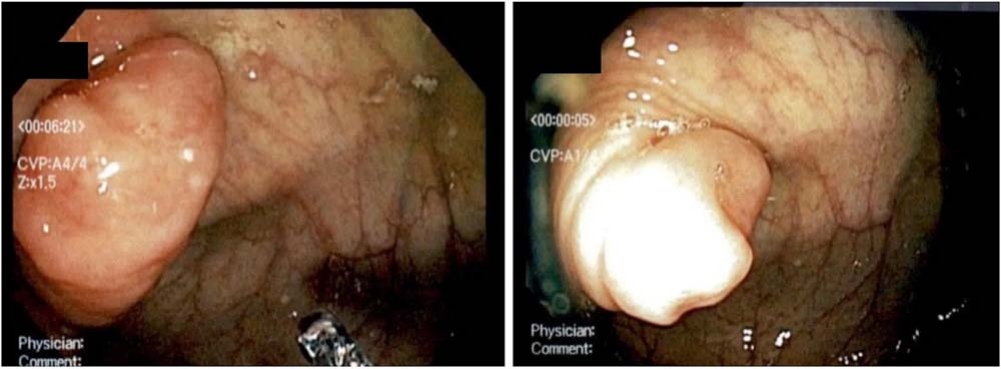
Screening colonoscopy showing inverted appendix.

**Figure 2:**
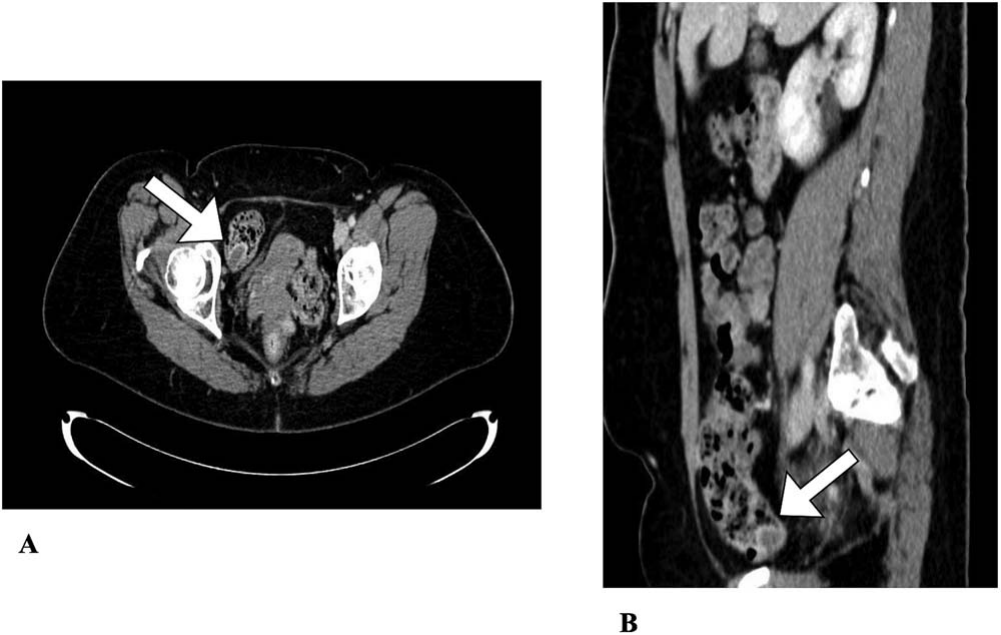
Abdominal CT. An appendiceal cecal intussusception is present (arrows) with the distally located appendix invaginating into the proximally located cecum. (A) Axial view. (B) Sagittal view.

A 52-year-old female was found to have an invaginated appendix on routine screening colonoscopy (Fig. 1). She underwent subsequent computed tomography (CT) which confirmed the appendix inverting into the cecum (Fig. 2). The patient presented with no acute appendicitis or abdominal symptoms. Due to the inability to rule out appendiceal neoplasm, she was provided treatment options including surveillance and surgical removal. The patient elected to have a robotic assisted appendectomy with possible ileocecal resection depending on severity of disease. Her medical history was significant for prior cesarean section, which was evident during the robotic procedure as the uterus was adhered to the lower abdominal wall. There was no visible appendix in the right iliac fossa during inspection, but tissue could be palpated within the distal cecum. During the procedure, it was determined that the ileocecal resection was not necessary. Instead, a partial cecectomy with appendectomy was performed to spare the ileocecal valve as it was not involved. Resected margins were 3.5 × 2.7 × 0.3 cm with the inverted appendix measuring 1.8 × 1.0 cm. The operation was performed without complications, and the patient was able to be discharged later that day. The postoperative period was without complications.

Histopathological examination revealed endometriosis of the appendix with no signs of malignancy. This was determined through immunohistochemistry utilizing CD10 to highlight endometrial stroma and PAX-8 to highlight epithelium.

## Discussion

This case presents a rare finding of appendiceal intussusception with concurrent appendiceal endometriosis. Intussusception of the bowel occurs predominantly in children, with adults comprising <5% of cases [4]. Of these, invagination of the appendix is a rare presentation in adults, with only 200 cases reported and >60% being attributable to neoplasms acting as lead points [1, 4]. Primarily, appendiceal intussusception is due to an inflammatory process (76%) and less often due to endometriosis (33%), further emphasizing the rarity of this finding [5].

Endometriosis is a condition of transplantation of endometrial tissues outside of the uterine cavity affecting 2%–10% of females [6]. Common sites involve the ovaries, fallopian tubes, and less frequently the gastrointestinal tract [7]. Possible explanations for endometriosis in the gastrointestinal tract or abdominal wall can be accounted for with previous gynecological surgeries as seen in our patient [7]. Most commonly, endometriosis implants on the rectosigmoid colon (70%–85%) followed by the terminal ileum (1.7%) and to a lesser extent the appendix and cecum [7].

Primary diagnosis of endometriosis of the appendix has been discovered due to mimicking acute appendicitis symptoms, cyclic right lower quadrant pain, and incidentally during gynaecological surgeries or colonoscopies [1, 2, 8]. In this case, our patient was asymptomatic. An inverted appendix was detected during her routine colonoscopy and a follow-up CT confirmed the diagnosis, but could not exclude a neoplasm. Therefore, discussion of a prophylactic appendectomy and partial cecectomy was recommended and our patient agreed to resection. Currently, there is no ‘Gold Standard’ of treatment for appendiceal intussusception secondary to endometriosis. Literature review shows that nearly all reported cases were managed by removing the affected tissues via appendectomy or partial colectomy as opposed to surveillance [5].

Most often, diagnosis of appendiceal endometriosis is made from pathological specimens acquired from biopsy or surgical specimens, making preoperative diagnosis difficult [9]. Histopathology of the appendix from our patient revealed endometrial tissues in the resected viscera. It is a common occurrence to find endometriosis incidentally rather than expected preoperatively [9]. Although rare, it is important to make the definitive diagnosis of a benign endometrial neoplasm in the setting of a prior cesarean section due to the possibility of transformation to malignancy [10].

## Conclusion

Appendiceal intussusception due to endometriosis acting as a lead point is a very rare finding that is difficult to diagnose preoperatively. Cases may present with symptoms that resemble common emergency room complaints such as right lower quadrant pain, or they may be asymptomatic and discovered incidentally, raising concern for neoplasm. It is important to consider appendiceal endometriosis as a differential diagnosis in female patients presenting with right lower quadrant abdominal pain or cyclic symptoms. The appearance of appendiceal intussusception as a possible neoplasm, highlights the importance to obtain biopsies or tissues from appendectomy to rule out malignancy and make a definitive diagnosis.
